# Clinical Application of a Multiplex Droplet Digital PCR in the Rapid Diagnosis of Children with Suspected Bloodstream Infections

**DOI:** 10.3390/pathogens12050719

**Published:** 2023-05-16

**Authors:** Wenxin Liu, Chun Wang, Fen Pan, Jingbo Shao, Yun Cui, Dingding Han, Hong Zhang

**Affiliations:** 1 Department of Clinical Laboratory, Shanghai Children’s Hospital, School of Medicine, Shanghai Jiao Tong University, Shanghai 200062, China; 2 Institute of Pediatric Infection, Immunity, and Critical Care Medicine, School of Medicine, Shanghai Jiao Tong University, Shanghai 200062, China; 3 Department of Hematology/Oncology, Shanghai Children’s Hospital, School of Medicine, Shanghai Jiao Tong University, Shanghai 200062, China; 4 Department of Critical Care Medicine, Shanghai Children’s Hospital, School of Medicine, Shanghai Jiao Tong University, Shanghai 200062, China

**Keywords:** droplet digital PCR (ddPCR), bloodstream infections (BSIs), blood culture (BC), children, viraemia

## Abstract

Droplet digital PCR (ddPCR) recently has been shown to be a potential diagnostic tool for adults with bloodstream infections (BSIs); however, its application in children remains obscure. In this study, 76 blood samples of children with suspected BSIs were synchronously detected by traditional blood cultures (BCs) and ddPCRs. Our team validated the diagnostic performance of ddPCR including sensitivity, specificity, and positive and negative predictive values. The 76 pediatric patients from the hematology department (67.1%), the pediatric intensive care unit (PICU, 27.6%), and other departments (5.2%) were enrolled. The positive rate of ddPCR results was 47.9%, whereas that for BC was 6.6%. In addition, the time consumption of ddPCR was shorter, only for 4.7 ± 0.9 h, in comparison with the detection timing of BC (76.7 ± 10.4 h, *p* < 0.01). The levels of agreement and disagreement between BC and ddPCR were 96.1% and 4.2%, and the negative agreement reached 95.6%. The sensitivity of ddPCR was 100%, with corresponding specificities ranging from 95.3 to 100.0%. In addition, a total of nine viruses were identified by ddPCR. In China, the multiplexed ddPCR first could be a tool for the rapid and accurate diagnosis of children with suspected BSIs and can be an early indicator of the possibility of viraemia in children with immunosuppression.

## 1. Introduction

Bloodstream infections (BSIs) account for morbidity and mortality in children worldwide. Sepsis has become a crucial global health problem, resulting in life-threatening organ dysfunction due to a dysregulated host response to infection [1]. Recently, nearly half of blood infections worldwide occurred in children; notably, almost 2.9 million children younger than five years have died from sepsis [2,3,4]. Globally, gram-negative bacteria accounted for a majority of pathogens in children with BSIs, followed by gram-positive bacteria and fungi [5,6,7]. Generally, the most common gram-negative bacterial isolates were *Klebsiella* spp., *Escherichia coli* (*E. coli*), *Acinetobacter baumannii* (*A. baumannii*), and *Pseudomonas aeruginosa* (*P. aeruginosa*), and the most frequent gram-positive bacterial isolates were *Staphylococcus aureus* (*S. aureus*), Group B *Streptococcus* (GBS), Coagulase-negative *Staphylococci* (CoNS), and *Enterococcus* spp. Similarly, in China, from the pathogens isolated from blood samples of children with BSIs, the largest proportion was gram-negative bacteria including *E. coli* and *Klebsiella pneumoniae* (*K. pneumoniae*), followed by gram-positive bacteria consisting of CoNS, *S. aureus*, and *Enterococcus* spp., and fungi [8,9]. The onset of BSIs in pediatric patients could be insidious and difficult to identify [10]. In addition, immunosuppression on the prognosis of children with BSIs had adverse effects [11]. Antimicrobial resistance and the inappropriate use of antibiotic drugs increase the burden of treatment in children with BSIs. It has been shown that every hour of delay in appropriate antimicrobial treatment would increase mortality by 7.6%, and a prompt diagnosis and treatment could prevent the increase in sepsis deaths [12]. In addition, previous studies have shown antimicrobial resistance (AMR) profiles of pathogens also caused neonatal sepsis; through genomic screening of genes coding for drug resistance, *bla_NDM_*, *bla_OXA-48_*, and *bla_KPC_* in gram-negative BSIs were considered as crucial factors, and *vanA* was considered as crucial in gram-positive BSIs [13,14]. Therefore, it is vital that an early and rapid diagnosis to clinically guide the appropriate administration of antibiotics relieves the pain of children with BSIs. Currently, the causative pathogen is identified by a traditional blood culture (BC), a gold standard, which will delay the optimal diagnosis time of BSIs due to long turnaround times and low sensitivity. Hence, the development of accurate, rapid, and sensitive diagnostic tools for the timely treatment of children with BSIs is an urgent matter.

In recent years, the rapid causative pathogen identification in the diagnosis of BSIs was prone to culture-independent, real-time polymerase chain reaction (PCR)-based, or microarrary-based methods, supplemental to conventional BC. In children, it has also been reported that current molecular approaches included a CE-marked multiplex real-time PCR diagnostic system directly from the blood, the LightCycler SeptiFast test [15], T2 magnetic resonance-based T2Candida and T2Bacteria panels [16], and plasma microbial cell-free DNA sequencing (mcfDNA-seq) [17]. Although these techniques could shorten the turnaround time to hours, there were several limitations such as medium sensitivity and specificity at low concentrations and the inability to synchronize antimicrobial susceptibility testings (ASTs). ddPCR, an emerging tool for rapid and sensitive pathogen identification used as a precise bedside test, has developed to overcome these challenges. In contrast to other molecular tests, the next-generation PCR method characterizes sensitivity, specificity, reproducibility, and absolute quantifications without a standard curve [18]. However, few studies have focused on the application of ddPCR for children with suspected BSIs.

In the present study, our team used ddPCR for the first time to detect causative pathogens and related resistance genes in children with suspected BSIs with a turnaround time of 4 h. On the basis of the latest data of China Antimicrobial Surveillance Network (CHINET) and the common pathogens isolated from our hospital, the designed ddPCR panel consisted of five panels that could identify five gram-positive bacteria (*Staphylococcus aureus*, Coagulase-negative *Staphylococcus*, *Enterococcus* spp., *Streptococcus* spp., and *Listeria monocytogenes*), nine gram-negative bacteria (*Pseudomonas aeruginosa*, *Enterobacter cloacae*, *Klebsiella* spp., *Escherichia coli*, *Acinetobacter baumannii*, *Salmonella* spp., *Bacteroides fragilis*, *Hemophilus influenzae*, and *Morganella* spp.), and one fungus (*Candida* spp.), as well as seven antimicrobial resistance genes (*bla_KPC_*, *mecA*, *OXA-48*, *NDM*, *IMF*, *vanA*, and *vanM*) and five herpes family viruses (herpes simplex virus-1, herpes simplex virus-2, varicella-zoster virus, cytomegalovirus, and Epstein–Barr virus). It is worth noting that for the first time in China, the combined application of viral panel and common pathogens and resistance markers achieved a rapidly comprehensive diagnosis of immunosuppressed children with suspected BSIs. In addition, the concordance and discordance between ddPCR and conventional BC results as well as diagnostics of ddPCR were evaluated.

## 2. Materials and Methods

### 2.1. Study Population and Sample Collection

**Figure 1:**
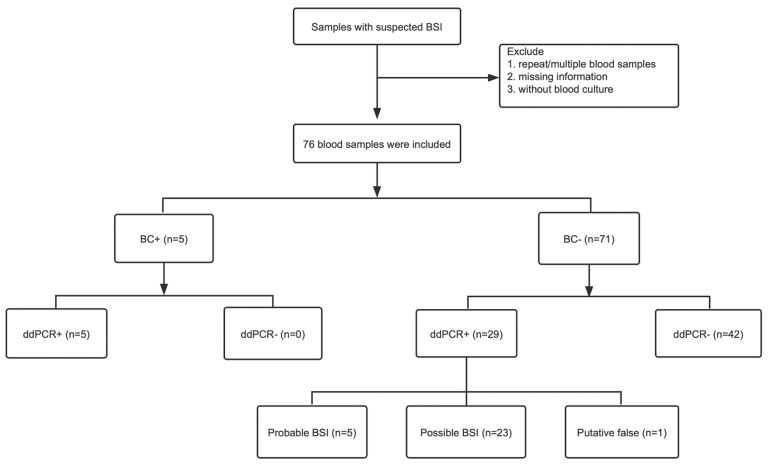
Flow chart of blood screening criteria and results analysis.

This study was the first to adopt the multiplex ddPCR as a diagnostic tool for the assessment of children with suspected BSIs in association with conventional BCs. The study was performed in the clinical lab of Shanghai Children’s Hospital, Shanghai Jiao Tong University School of Medicine, from 5 August 2022 to 20 December 2022. The enrolled pediatric patients with suspected BSIs were all younger than 18 years old, mostly from the hematology department and the PICU. The exclusion criteria were as follows (Figure 1): (1) patients in whom a simultaneous blood culture was not obtained in addition to the EDTA blood sample were not included in the study; (2) blood samples with inadequate clinical information or missing experimental data were ruled out; and (3) for the patients tested multiple times during hospitalization, only the first results were retained. Upon the clinical suspicion of a BSI, whole blood was drawn synchronously from the same vein or central venous line for the BC and the molecular diagnosis. According to hospital practices and international recommendations for pediatric populations [19,20,21], the BCs collected one bottle inoculated with 1.5–3 mL whole blood for each pediatric patient. An amount of 1–1.5 mL whole blood inoculated into an ethylenediaminetetraacetate (EDTA) blood collection tube was used for ddPCR detection.

### 2.2. Blood Culture and Pathogen Identification

The collected blood culture bottles were incubated at 37 °C in a BACTEC^TM^ FX200 (Becton, Dickinson, ND, USA). When a positive signal was reported by the system, gram staining and isolation culturing of the samples were performed simultaneously. The cultured isolates were further identified by matric-assisted laser desorption ionization-time of flight-mass spectrometry (MALDI-TOF MS; Bruker Daltonik GmbH, Bremen, Germany).

### 2.3. Plasma DNA Extraction and ddPCR Testing 

**Figure 2:**
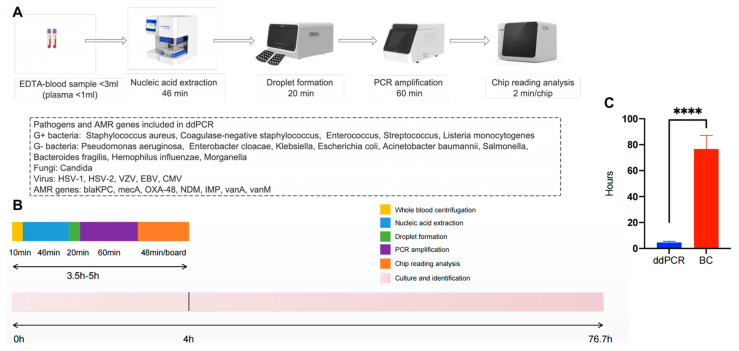
The detection process and time of droplet digital PCR. (**A**) ddPCR detection process and detection target. (**B**) Timing for ddPCR testing relative to BC. (**C**) Comparison between the detection time of ddPCR panel and BC. EDTA, ethylenediamine tetraacetic acid; AMR genes, antimicrobial resistance genes; HSV-1, herpes simplex virus-1; HSV-2, herpes simplex virus-2; VZV, varicella zoster virus; EBV, Epstein–Barr virus; CMV, cytomegalovirus. ****, *p* < 0.0001.

Upon receipt of the whole blood samples, they were centrifuged at 1200× *g* for 10 min immediately. Plasma DNA was extracted by a Magnetic Plasma DNA Kit within 40 min according to the manufacturer’s protocol (Pilot Gene Technologies, Hangzhou, China) (Figure 2). DNA was extracted from the 50 μL of elution buffer for subsequent use. The multiplex ddPCR testing based on 15 pathogens and seven antibiotic resistance genes identified using a Digital PCR Detection Kit (Cat. No. 4201008) (Pilot Gene Technologies, Hangzhou, China), which consists of five assay panels with five channels, detected pathogens and AMR genes including fourteen bacteria, one fungus, seven AMR genes, and five viruses. The description of the kit and the user’s manual can be found at www.pilotgene.com. Our team applied the Pilot Gene Droplet Digital PCR System to perform ddPCR analysis. Five μL of isolated plasma DNA was added to fifteen μL ddPCR master mix, which additionally contained four μL primer probe, three μL 5 × aTaq Mix, two μL ddH_2_O, and one μL internal reference. The reaction mixture in the sample cup was passed through the micro-channel (Droplet Generator DG32) under the action of pressure, and tens of thousands of water-in-oil emulsion droplets were generated due to gravity and shear force in 20 min [22]. Next, Thermal Cycler TC1 performed PCR amplification for 60 min and then using a chip scanner CS5 and GenePMS software (v2.0.01.20011) scanned and analyzed the data for droplet counts and amplitudes. The synthesized DNA fragments served as the positive controls, and DNase-free water served as the negative control to eliminate external or reagent microbial contamination. In addition, each batch of kits was used for the first time with a positive control of the standard product to rule out false negatives. The procedure was the same as above except the plasma was replaced with the positive control of the standard product. Noteworthily, each panel included an internal control as a reference and was verified through a cross-validation test (Supplementary Material). The results of ddPCR were presented as the copies of each targeted pathogen or gene. When one or more ddPCR-targeted pathogens were detected, the ddPCR results were considered positive, while none were detected to be negative. When the BC results were negative, we could divide the results of ddPCR into three classifications: (1) probable BSI, where the ddPCR results were consistent with the culture results of other sites within seven days; (2) possible BSI, where in combination with laboratory examinations and a clinical diagnosis, the ddPCR results without microbiological data showed potential pathogenicity; and (3) putative false, where the ddPCR results were discordant with the clinical evidence.

### 2.4. Statistical Analysis

IBM SPSS Statistics software (v 26.0) (IBM, Armonk, NY, USA) was used for database management and statistical analysis. Continuous variables were expressed as the median and interquartile range (IQR). The normally distributed continuous variables were analyzed by the *t* test, while the nonnormally distributed continuous variables were analyzed by the Mann–Whitney *U* test. The chi-square was used to analyze categorical variables, which were expressed as frequencies and percentages. MedCalc, version 20.010 (MedCalc Software, Ostend, Belgium), was used to calculate the sensitivity, specificity, and positive and negative predictive values.

## 3. Results

### 3.1. Clinical Characteristics of Recruited Patients

Taking exclusion/inclusion criteria into consideration, a total of 76 patients with suspected BSIs were recruited in this study (Table 1). Among them, the median age of the patients was 6.1 ± 3.8 years, and there were 39 males (51.3%). The patients were predominantly recruited from the hematology department and the PICU (51 patients and 21 patients, respectively), and others were recruited from the neonatology department and the pneumology department. Most of these children had multiple comorbidities, including 38 children (50%) with hematological malignancies, 17 children (22.4%) with respiratory failure, 10 children (13.2%) with gastrointestinal dysfunction, 13 children (17.1%) with coagulation disorders, and 12 children (15.8%) with anemia. In terms of inflammatory markers, the median plasma levels of C-reactive protein and procalcitonin were 26.0 mg/L (IQR, 6.0–81.0) and 0.2 μg/L (IQR, 0.1–1.1), respectively. From the perspective of coagulation function, the levels of fibrinogen and D-dimer were 4.1 mg/dL (IQR, 2.5–5.4) and 1.1 μg/L (IQR, 0.5–2.2), respectively. In addition, the hemoglobin level of the children was 85 g/L (IQR, 74–106).

### 3.2. Performance of the Blood Culture Testing

**Figure 3:**
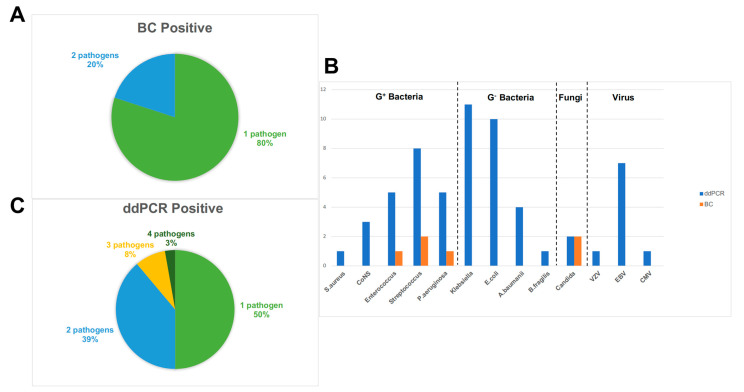
Pathogens detected by BC and ddPCR. (**A**) Counts of pathogens infected by blood culture-positive patients. (**B**) Pathogens detected by ddPCR and BC within the detection range of ddPCR. (**C**) Counts of pathogens infected by ddPCR-positive patients. G+ bacteria, gram-positive bacteria; G- bacteria, gram-negative bacteria; BC, blood culture; *S. aureus*, *Staphylococcus aureus*; CoNS, coagulase-negative *Staphylococcus*; *P. aeruginosa*, *Pseudomonas aeruginosa*; *E. coli*, *Escherichia coli*; *A. baumannii*, *Acinetobacter baumannii*; *B. fragilis*, *Bacteroides fragilis*; VZV, varicella zoster virus; EBV, Epstein–Barr virus; CMV, cytomegalovirus.

Within the range of ddPCR-targeted organisms, a total of 1140 microorganisms were detected in 76 children; meanwhile, blood culture also detected positive results for six microorganisms in five blood samples (Figure 1). Among the six positive blood cultures, two microorganisms were fungi, including two strains of *C. tropicalis*; three microorganisms were gram-positive bacteria, including two strains of *S. mitis*/*S. oralis* and one strain of *E. faecium*; and one microorganism was a *P. aeruginosa* strain belonging to gram-negative bacteria (Table 2). In addition, the cases of polymicrobial infection detected by blood culture accounted for 20% (Figure 3A).

### 3.3. Pathogens and AMR Genes Detected by ddPCR

In total, 1140 microorganisms of blood samples from 76 children within the ddPCR target range were detected, with 50 positive ddPCR results (Table 3). Among 50 pathogens, we detected 17 gram-positive bacteria, mostly including *Streptococcus* spp. (*n* = 8) and *Enterococcus* spp. (*n* = 5); 31 gram-negative bacteria, with the top three strains being *Klebsiella* spp. (*n* = 11), *E. coli* (*n* = 10), and *P. aeruginosa* (*n* = 5); and two fungi, such as *Candida* spp. Significantly, our team created a viral panel and detected nine pathogens in 76 blood samples including EBV (*n* = 7), VZV (*n* = 1), and CMV (*n* = 1, Figure 3B, Table 4). In addition, ddPCR detecting polymicrobial infections was 50% (Figure 3C).

In our study, we used the AMR panel to detect seven AMR genes including *bla_KPC_*, *mecA*, *OXA-48*, *NDM*, *IMP*, *vanA*, and *vanM*; however, only the *bla_KPC_*, *mecA*, and *OXA-48* genes were detected as positive by ddPCR testing (Table 5). The *bla_KPC_* gene together with *E. coli* was identified in the blood sample. Strangely, *P. aeruginosa* and *E. coli* were synchronously detected in a mecA-positive sample. In addition, no pathogen was tested in the OXA-48 positive sample.

### 3.4. Comparison between ddPCR and Blood Culture

In this study, within the detection range of ddPCR-targeted organisms, 50 pathogens were detected by ddPCR, whereas only six pathogens were detected by blood culture (Table 4). The ddPCR results were positive in 100% (6/6) of microorganisms with positive BCs. Compared with the negative results of BCs, 44 pathogens were positive in ddPCR tests. The 44 discordant microorganisms included *Klebsiella* spp. (*n* = 11), *E. coli* (*n* = 10), *Streptococcus* spp. (*n* = 6), *Enterococcus* spp. (*n* = 4), *P. aeruginosa* (*n* = 4), *A. baumannii* (*n* = 4), CoNS (*n* = 3), *S. aureus* (*n* = 1), and *B. fragilis* (*n* = 1). In addition, the average detection time of the ddPCR panel was 4.7 ± 0.9 h, which was far shorter than that of the blood culture (76.7 ± 10.4 h, *p* < 0.01; Table 1 and Figure 2C).

The results of ddPCR and BCs remained consistent, with six microorganisms being concordantly positive and 1090 microorganisms being concordantly negative (Table 6). The level of agreement between BCs and ddPCR was 96.1% (1096/1140), in which the positive agreement was 0.5% (6/1140), while the negative agreement reached 95.6% (1090/1140). On the basis of BC testing, the calculation principle was the aggregate ddPCR detection, demonstrating a sensitivity of 100.0%, a specificity of 96.1%, a positive predictive value (PPV) of 12.0%, and a negative predictive value (NPV) of 96.1%. Specifically, the sensitivity of ddPCR-targeted gram-positive/negative bacteria and fungi was 100.0%. In addition, ddPCR-targeted gram-positive/negative bacteria and fungi were highly specific, with 95.3%, 96.0%, and 100%, respectively.

## 4. Discussion

Globally, bloodstream infections (BSIs) are the primary cause of morbidity and mortality among children. Blood culture remains the gold standard for the laboratory diagnosis of BSIs. It is worth noting that blood volume is a critical factor for successful pathogen recovery during a blood culture [23]. In addition, the inoculated blood volume also determines the sensitivity and specificity of blood culture [24]. For adults, the recommended standard blood volume per BC bottle is 8–10 mL, and two sets are usually taken containing one aerobic and one anaerobic BC bottle [19,25]. However, the optimal inoculated blood volume in pediatric patients remains controversial. Huber et al. [26] has summarized the opinions of multiple recent studies and graded the blood volume based on self-defined age and weight. The ages of pediatric patients were divided into >1 y, ≥1–3 y, >3–10 y, and ≥10 y, and the corresponding blood volumes were >0.5–3.0 mL, 1.0–4.0 mL, 3.0–8.0 mL, and 20.0 mL, respectively [27,28,29,30]. The patient weights were divided into ≤2.0 kg, >2.0–5.0, >5.0–10.0, >10.0–20.0, and >20.0–30.0, and the corresponding blood volumes were 1.0–4.5 mL, 1.0–6.0 mL, 1.5–10.0 mL, 6.0–23.0 mL, and ≥10.0 mL, respectively [19,25,31,32,33,34]. Compared with BCs, the ddPCR protocol usually required less blood volume (1–1.5 mL) while having a higher positive rate of ddPCR results, demonstrating the characteristics of high sensitivity. Furthermore, the small inoculated blood volume in pediatric patients can reduce the occurrence of painful events, dramatically increase the children’s level of cooperation, and increase the convenience and speed of blood collection by medical staff. In addition, the ddPCR panel provided definitive microorganism identification in an average time of four hours, whereas the detection timing of BC was relatively longer (Figure 2B).

A previous study has suggested that even if proper aseptic BC procedure and methodology are carried out, there will be 1–3% BC contamination rate [26]. In addition, inadequate blood volume inoculation into culture bottles will increase the risk of culture contamination, resulting in unnecessary antimicrobial treatment for children suspected of a BSI and missing the optimal treatment time, which causes serious adverse consequences. Therefore, it is urgent to select a novel technology with characteristics of low contamination, short time consumption, and easy sample collection. Given the extreme sensitivity of ddPCR, even minimal environmental or sample contamination would be evidenced with this method. So, in such a case, thorough disinfection of working environment and instruments is necessary. In our study, 34 blood samples (47.9%) were positive for ddPCR in comparison with five positive samples (6.6%) in BCs, demonstrating that ddPCR had potential advantages over BCs. A previous study has demonstrated, compared with positive preantimicrobial cultures, the sensitivity of blood cultures obtained after empirical treatment decreased approximately 50%, and noteworthily, the reduction in sensitivity remained meaningful for microbiological cultures in other anatomical sites [35]. The low positive rate of BC results might be attributed to the administration of empirical antimicrobial therapy performed by clinicians in children with suspected BSIs within 72 h prior to sampling (Table S1). Furthermore, the observation of a positive rate for BCs in our study was similar to a previous report. In that study, the overall rate of positive blood cultures in children was low, with the positive rate only 5.2% for adequate blood culture volume, which was defined as ≥0.5 mL blood culture volume for patients <1 month of age, ≥1.0 mL for patients <36 months of age, and ≥4.0 mL for patients ≥36 months, while it was merely 2.1% for inadequate blood culture volume [27]. It was almost inevitable that a large proportion of blood cultures would be negative because of the submission of an inadequate volume of blood in children compared with adults [27]. Among the 76 pediatric patients, ddPCR detected three panels containing bacteria and fungi, with five pathogens on each panel to be detected; therefore, a total of 1140 microorganisms were detected within the range of the ddPCR-targeted organism. In our study, the BC+/ddPCR+ and BC-/ddPCR- results showed six and 1090 of the 1140 microorganisms of ddPCR-targeted pathogens, respectively. In other words, there were 1096 (96.1%) concordant positive or negative results between the two methods. Furthermore, six microorganisms with positive BCs were also synchronously detected by ddPCR, with either gram-positive/negative bacteria or fungi, with a sensitivity of 100%. The corresponding specificity ranged from 95.3% in G+ bacteria to 100% in fungi, with an aggregate specificity of 96.1%. In addition, there were 44 discordant BC−/ddPCR+ results; from the perspective of detailed clinical circumstances, the majority of the discordant results were either probable or possible BSIs (Figure 1 and Table S1). This might be explained by the presence of nonviable, nonproliferating, or transient or intermittent bacteremia, intracellular organisms within circulating phagocytic cells, inhibition of bacterial growth by antibiotics, or possible contamination [22,36]. Therefore, it is necessary to take ddPCR as a supplementary method to conventional BCs to identify the possible causative pathogens for BC-negative pediatric patients. In our study, although ddPCR detection among children with suspected BSIs has the characteristics of high sensitivity, good specificity, and speed, to a certain extent, there are some limitations in the detection of AMR genes. We only detected *bla_KPC_*, *mecA*, and *OXA-48*, and only *bla_KPC_* might be carried by E. coli, while no proper causative pathogens were identified for the other two AMR genes. The emergence of the plasmid gene *OXA-48* might be due to the different stabilities between bacterial cfDNA and cell-free plasmids as previously reported [37,38]. The secondary structure may have played an important role in protecting the plasmid DNA from nuclease degradation compared with the linear genomic DNA. However, gene *mecA* is usually located in the *Staphylococcal* chromosome. Given the AMR gene identified by ddPCR detection is not from the isolated pathogen strain, which is different from the traditional culture-based method, the cause of the mismatch between the pathogen and AMR genes needs to be thoroughly investigated in the future. Obviously, the application of ddPCR to detect AMR genes in pediatric patients with BSIs needs further design, optimization, and verification of this panel.

Recently, in addition to ddPCR, there are some rapid molecular diagnostic methods that can directly detect multiple pathogens and resistance phenotypes in the whole blood without cultivating organisms. A previous study has shown that metagenomic next-generation sequencing (mNGS) of plasma cell-free DNA has become a powerful tool for identifying the pathogens responsible for BSIs [39]. mNGS detected a wider spectrum of pathogens and was more suitable for diagnosing rare infections and intractable diseases [22,40]. However, some common causative pathogens were occasionally missing during the detection of mNGS, in comparison with conventional BCs [41,42]. In addition, within the range of ddPCR-targeted pathogens, the positive rate of mNGS was lower than the ddPCR assay [40]. Considering economic factors, the cost of mNGS is considerably higher than that of the ddPCR assay. On the other hand, in the pediatric population, T2 magnetic resonance technology is expected to be an effective rapid diagnostic tool for bacterial and fungal bloodstream infections [16]. T2Bacteria and T2Candida panels have the characteristics of high sensitivity, good specificity, short time consumption, and less blood volume collection. Furthermore, the T2Resistance^®^ panel can detect thirteen common AMR genes including *bla_KPC_*, *OXA-48*, and *mecA* [43]. Nevertheless, the panel of T2 magnetic resonance technology lacks the detection of viruses; in addition, recently, few clinical trials have been conducted, so whether it is really appropriate for the clinical diagnosis of children with BSIs needs to be further investigated.

Owing to the clear predominance of bacterial and fungal infections in the context of children with BSIs, screening for viral infections is rarely part of routine diagnostics. A previous study showed the viral DNAemia was commonly found in the plasma of children with severe sepsis; in addition, pediatric patients with pre-existing immune suppression were at the greatest risk for viral DNAemia [44]. They used real-time polymerase chain reaction (RT-PCR) to detect CMV and EBV in septic children with the pre-existing immune suppression. Similar to our results, we used ddPCR to detect CMV and EBV in suspected BSIs in children with previous immunosuppressive diseases such as lymphocytic leukemia, granulocytic leukemia, and Hodgkin’s lymphoma. For the first time in a children’s hospital, our team designed a novel panel for the herpes family viruses, including HSV-1, HSV-2, VZV, EBV, and CMV, in the range of ddPCR-targeted pathogens. We only detected VZV, EBV, and CMV, and the positive rate of EBV was significantly higher than the other two viruses. Similarly, previous studies have shown that EBV was the most commonly observed reactivated virus in plasma in septic patients [45,46,47]. EBV, as the first human tumor virus expressing virus cancer genes and immortalizing infected lymphocytes, resides in humans to establish a long-term latent infection and is associated with a variety of human diseases including hematologic malignancies [48]. In children, the spectrum of EBV-associated lymphoid malignancies is expanding from Burkitt lymphoma to the systemic EBV^+^ T cell lymphoproliferative disease [49]. In this study, more than 50% of 76 pediatric patients with suspected BSIs accompanied hematologic malignancies and malignant tumors. The levels of viraemia might be considered to be a useful biomarker of immunosuppression, guiding immunotherapy and monitoring disease progression and response to therapy [11]. As already known, quantitative real-time polymerase chain reaction (qPCR) and reverse transcription qPCR (RT-qPCR) are currently used for pathogenic viruses detection, but it is common to overestimate the number of infectious viruses during the process of detecting viral nucleic acids [50]. In comparison with qPCR, ddPCR could estimate the absolute quantification of viruses more sensitively and accurately without standard curves. In addition, mNGS, as a single universal virus detection method, has several hurdles that need to be addressed such as time consumption, extreme cost, insensitivity method standardization and data storage, protection, analysis, and interpretation compared with ddPCR [51]. If this were the case, ddPCR, an efficient and sensitive assay, can be used to rapidly detect children with immunosuppressive disorders to monitor or rule out the risk of viraemia in the future. Apart from hematologic malignancies and malignant tumor, sepsis also caused acidaemia and hypoxia leading to respiratory failure and ARDS [52]. In addition, previous studies have shown the association between thyroid function and bloodstream infection, with hypothyroidism related to higher mortality of sepsis [53,54,55]. DIC, a frequent serious complication of sepsis, threatens the health and life of children [56,57]. Sepsis-associated DIC characterizes an abnormal state of coagulation and excessive suppression of the fibrinolytic system, resulting in microthrombosis, reduced tissue perfusion, and multiple-organ dysfunction [58]. Generally, pediatric sepsis-related complications aggravate clinical treatment difficulties. Therefore, the rapid identification of pathogens through the ddPCR panel plays a crucial role in delaying the occurrence of complications. However, whether sepsis-related hemodynamic changes would affect the results of ddPCR panels remains obscure and needs to be further studied.

Several limitations need to be mentioned in this study. First, detections of viruses and AMR genes were not simultaneously compared with other molecular tests, so the results of virus and AMR genes in this study need further investigation. In addition, we only compared the results of ddPCR with conventional BC results, so multiple detection methods should be added to make the results more meaningful. Second, there was no in-depth analysis of quantitative ddPCR for the relationship between the pathogen load detected and the infection severity. Finally, because of the small volume of blood in children, the remaining blood volume or the extracted DNA volume after the genus level is insufficient for further species level testing.

## 5. Conclusions

Our team was the first to use the multiplex ddPCR to detect blood samples with suspected BSIs in pediatric patients. In the near future, ddPCR, a promising and potential detection method to rapidly and accurately identify causative pathogens, can be used to replenish conventional BC methods in the children’s hospital. The newly added viral panel is also helpful for the early diagnosis of viraemia in children with immunosuppression. The AMR gene panel should be redesigned and revalidated before ddPCR can be used to diagnose children with suspected BSIs.

## Figures and Tables

**Table 1:** Clinical characteristics of recruited patients.

Clinical Characteristics	*n* = 76
Age, years	6.1 ± 3.8
Male, *n* (%)	39 (51.3)
Departments	
Hematology department, *n* (%)	51 (67.1)
PICU, *n* (%)	21 (27.6)
Neonatology department, *n* (%)	3 (3.9)
Pneumology department, *n* (%)	1 (1.3)
Comorbidities	
Hematological malignancies, *n* (%)	38 (50)
Malignant tumor, *n* (%)	8 (10.5)
Gastrointestinal dysfunction, *n* (%)	10 (13.2)
Respiratory failure, *n* (%)	17 (22.4)
ARDS, *n* (%)	2 (2.6)
Virus infection, *n* (%)	6 (7.9)
Hypothyroidism, *n* (%)	5 (5.6)
Anemia, *n* (%)	12 (15.8)
Coagulation disorders, *n* (%)	13 (17.1)
DIC, *n* (%)	3 (3.9)
Laboratory examination	
Red blood cell count, median (IQR) × 1012/L	3.0 (2.4, 3.4)
Hemoglobin (g/L), median (IQR)	85 (74, 106)
White blood count, median (IQR) × 109/L	2.5 (0.6, 5.8)
Neutrophil count, median (IQR) × 109/L	0.5 (0.1, 3.2)
Lymphocyte count, median (IQR) × 109/L	0.9 (0.3, 1.6)
Platelet count, median (IQR) × 109/L	123.0 (40.0, 252.0)
C-reactive protein (mg/L), median (IQR)	26.0 (6.0, 81.0)
Procalcitonin (μg/L), median (IQR)	0.2 (0.1, 1.1)
Fibrinogen (mg/dL), median (IQR)	4.1 (2.5, 5.4)
D-dimer (μg/L), median (IQR)	1.1 (0.5, 2.2)

PICU, pediatric intensive care unit; ARDS, acute respiratory distress syndrome; DIC, disseminated intravascular coagulation.

**Table 2:** Comparison of pathogen detection among the ddPCR and BC methods in BC-positive patients.

Sample Number	Blood Culture	ddPCR
9	*C. tropicalis*	*Candida* spp. *A. baumannii*
11	*C. tropicalis*	*Candida* spp. *E. coli*
23	*E. faecium* *S. mitis/S. oralis*	*Enterococcus* spp. *Streptococcus* spp. *P. aeruginosa* *Klebsiella* spp.
54	*S. mitis/S. oralis*	*Streptococcus* spp.
63	*P. aeruginosa*	*P. aeruginosa*

*C. tropicalis*, *Candida tropicalis*; *A. baumannii*, *Acinetobacter baumannii*; *E. coli*, *Escherichia coli*; *E. faecium*, *Enterococcus faecalis*; *S. mitis*/*S. oralis*, *Streptococcus mitis*/*Streptococcus oralis*; *P. aeruginosa*, *Pseudomonas aeruginosa*.

**Table 3:** Comparison between BC and ddPCR results within the range of ddPCR-targeted organisms.

	BC^+^/ddPCR^+^, *n*	BC^+^/ddPCR^−^, *n*	BC^−^/ddPCR^+^, *n*	BC^−^/ddPCR^−^, *n*
All pathogens	6	0	44	1090
*Candida* spp.	2	0	0	-
*Enterococcus* spp.	1	0	4	-
*Streptococcus* spp.	2	0	6	-
*P. aeruginosa*	1	0	4	-
*Klebsiella* spp.	0	0	11	-
*E. coli*	0	0	10	-
*S. aureus*	0	0	1	-
CoNS	0	0	3	-
*B. fragilis*	0	0	1	-
*A. baumanii*	0	0	4	-

*S. aureus, Staphylococcus aureus*; CoNS, coagulase-negative *Staphylococcus*; *P. aeruginosa*, *Pseudomonas aeruginosa*; *E. coli*, *Escherichia coli*; *A. baumannii*, *Acinetobacter baumannii*; *B. fragilis*, *Bacteroides fragilis*.

**Table 4:** Viral pathogens detected by the ddPCR panel. VZV, varicella zoster virus; EBV, Epstein–Barr virus; CMV, cytomegalovirus.

Sample Number	Virus
1	VZV
20	EBV
36	EBV
44	EBV
58	EBV, CMV
63	EBV
70	EBV
73	EBV

**Table 5:** AMR genes detected by ddPCR.

Sample Number	AMR Gene	Pathogens
13	*OXA-48*	None
11	*bla_KPC_*	*E. coli*
25	*mecA*	*P. aeruginosa* *E. coli*

AMR gene, antimicrobial resistance gene; *E. coli*, *Escherichia coli*; *P. aeruginosa*, *Pseudomonas aeruginosa*.

**Table 6:** Comparison of positive and negative agreement of ddPCR and BC.

	Sample (*n* = 1140)	BC^+^	BC^−^	Sensitivity (%)	Specificity (%)	PPV (%)	NPV (%)
Total	ddPCR^+^	6	44	100.0 (54.0, 100.0)	96.1 (94.8, 97.2)	12.0 (9.3, 15.4)	96.1
	ddPCR^−^	0	1090				
G^+^ bacteria	ddPCR^+^	3	14	100.0 (29.2, 100.0)	95.3 (92.3, 97.4)	17.6 (11.4, 26.3)	100.0
	ddPCR^−^	0	287				
G^−^ bacteria	ddPCR^+^	1	30	100.0 (2.5, 100.0)	96.0 (94.4, 97.3)	3.2 (2.3, 4.5)	100.0
	ddPCR^−^	0	729				
Fungi	ddPCR^+^	2	0	100.0 (15.8, 100.0)	100.0 (95.1, 100.0)	100.0	100.0
	ddPCR^−^	0	74				

G^+^ bacteria, gram-positive; G^−^ bacteria, gram-negative bacteria; BC, blood culture; PPV, positive predictive value; NPV, negative predictive value.

## Data Availability

The original contributions presented in the study are included in the article/Supplementary Material. Further inquiries can be directed to the corresponding authors.

## Supplementary Materials

The following supporting information can be downloaded at: https://www.mdpi.com/article/10.3390/pathogens12050719/s1, Table S1: Detailed clinical information of BC-/ddPCR+ samples.
