# Supplementary material for: Clinical Application of a Multiplex Droplet Digital PCR in the Rapid Diagnosis of Children with Suspected Bloodstream Infections
Source: Pathogens. 2023 May 16;12(5):719. doi: 10.3390/pathogens12050719 (PMC10223642; doi:10.3390/pathogens12050719)
Supplement: Supplementary file 1 [file pathogens-12-00719-s001.zip › pathogens-2314111-supplementary.pdf]

Supplementary Table S1. Detailed clinical information of BC^{-}/$ddPCR^{+}$ samples.

Number	ddPCR results	Anti-bacterial agents	Culture results of other sites	Clinical diagnosis	Laboratory examination
**Probable BSI**					
17	*Klebsiella*	Ceftazidime<br>Itraconazole<br>Sulfamethoxazole (SMZ)<br>Caspofungin<br>Isopamicin<br>Sulperazon<br>Vancomycin<br>Tigecycline<br>Voriconazole	*Klebsiella pneumoniae* (endotracheal tube)<br>*CRKP* (faeces)	Acute hemorrhagic necrotizing pancreatitis<br>Severe pneumonia<br>Acute lymphocytic leukemia (ALL)	WBC (10^9/L):0.39<br>CRP (mg/L):139<br>PCT (μg/L): <0.05
20	*Streptococcus*<br>*EBV*	Meropenem<br>Vancomycin	*Methicillin-resistant Staphylococcus aureus* (*MRSA*, throat swab)<br>*C. albicans* (sputum)<br>*S. aureus* (sputum)<br>*S. anginosus* (hydrothorax)<br>*E. coli* (faeces)	Sepsis<br>Septic shock<br>Pyopneumothorax<br>Severe pneumonia	WBC (10^9/L):10.15<br>CRP (mg/L): >170<br>PCT (μg/L):27.52
53	*E. coli*	Sulperazon<br>Vancomycin	*Enterobacteriaceae* (faeces)<br>*Enterococcus* (faeces)	Sepsis<br>Bacterial pneumonia<br>Hepatoblastoma	WBC (10^9/L):1.08<br>CRP (mg/L): 88<br>PCT (μg/L): 0.16
59	*Klebsiella*	Sulperazon	*E. asburiae* (pus)	Sepsis	WBC (10^9/L):0.24

	Streptococcus<br>*CoNS*	Vancomycin	*S. viridans* (throat swab)	Bronchopneumonia<br>Retinoblastoma	CRP (mg/L): 57<br>PCT (μg/L): 0.37
62	*Klebsiella*	Sulperazon<br>Vancomycin<br>Fluconazole	*Klebsiella pneumoniae* (oral fluid)<br>*Enterobacteriaceae* (faeces)<br>*Enterococcus* (faeces)	Sepsis<br>Intestinal infection<br>ALL	WBC (10^9/L):0.43<br>CRP (mg/L): 98<br>PCT (μg/L): 2.91
**Possible BSI**					
1	*Bacteroides fragilis*<br>*VZV*	Meropenem<br>Vancomycin<br>Itraconazole	*Proteobacteria* (faeces)<br>*C.albicans* (faeces)	Neuroblastoma<br>Respiratory tract infection （RTI）<br>Autoimmuneencephalitis (AE)	WBC (10^9/L):0.46<br>CRP (mg/L):123<br>PCT (μg/L):3.85
2	*CoNS*	Isopamicin<br>Levofloxacin<br>Itraconazole	*P. Aeruginosa* (sputum, endotracheal intubation)	Medulloblastoma<br>Pneumonia<br>Central respiratory failure	WBC (10^9/L):3.27<br>CRP (mg/L):6<br>PCT (μg/L): <0.02
8	*Klebsiella*<br>*E. coli*	Meropenem<br>Vancomycin	*P. Aeruginosa* (hydrocele)	Epididymitis	WBC (10^9/L):39.79<br>CRP (mg/L):60<br>PCT (μg/L):2.26
16	*A. Baumanii*<br>*CoNS*	Isopamicin<br>Imipenem and Cilastatin Sodium<br>Linezolid<br>Voriconazole<br>Tigecycline	*Carbapenem-resistant Klebsiella pneumoniae* (CRKP, faeces)<br>*P. Aeruginosa* (faeces)	Sepsis<br>Pneumonia<br>Acute monocytic leukemia (AML)	WBC (10^9/L):0.27<br>CRP (mg/L):81<br>PCT (μg/L):0.06
21	*Streptococcus*	Isopamicin<br>Sulperazon	*Enterobacteriaceae* (faeces)<br>*Enterococcus* (faeces)	Sepsis<br>Septic shock	WBC (10^9/L):16.9<br>CRP (mg/L): 104

		Tigecycline<br>Fluconazole	*A. Baumanii* (sputum)	Osteosarcoma	PCT (μg/L):0.74
26	*Klebsiella*<br>*E. Coli*<br>*Enterococcus*	Vancomycin<br>Sulperazon<br>Isopamicin<br>Fluconazole	*S. viridans* (throat swab)<br>*A. Baumanii* (wound fluid)<br>*Enterobacteriaceae* (faeces)<br>*Enterococcus* (faeces)	Sepsis<br>T lymphocyte lymphoma<br>Stomatitis	WBC (10^9/L):2.38<br>CRP (mg/L):68<br>PCT (μg/L):0.87
29	*E. Coli*	Meropenem<br>Vancomycin	*Enterococcus* (faeces)<br>*E. coli* (faeces)	Sepsis<br>Purulent meningitis<br>Pneumonia	WBC (10^9/L):8.78<br>CRP (mg/L):153<br>PCT (μg/L):55.97
30	*E. coli*	Amoxicillin and clavulanate potassiun	-	Bronchopnumonia	WBC (10^9/L):17.95<br>CRP (mg/L):6<br>PCT (μg/L): <0.02
34	*E. coli*	Sulperazon<br>Cefurxim	-	Systemic inflammatory response syndrome (SIRS)<br>Acute lymphadenitis	WBC (10^9/L):5.43<br>CRP (mg/L):27<br>PCT (μg/L): 0.22
35	*P. Aeruginosa*<br>*Enterococcus*	Sulperazon<br>Ornidazole<br>Caspofungin<br>SMZ<br>Meropenem<br>Voriconazole	*Haemophilus parainfluenzae* (throat swab)<br>*S. viridans* (oral fluid)<br>*Neisseria* (oral fluid)	ALL	WBC (10^9/L):0.18<br>CRP (mg/L): <5<br>PCT (μg/L): 0.03
37	*P. Aeruginosa*<br>*E. coli*	Sulperazon<br>Fluconazole	*S. epidermidis* (throat swab)	Pneumonia<br>ALL	WBC (10^9/L):2.58<br>CRP (mg/L):25<br>PCT (μg/L): 0.14
41	*A. Baumanii*	Isopamicin	-	Intestinal infection	WBC (10^9/L):0.46

	Streptococcus	Sulperazon<br>Fluconazole<br>Vancomycin<br>Imipenem<br>SMZ<br>Imipenem and Cilastatin Sodium		Skin soft-tissue infection	CRP (mg/L):41<br>PCT (μg/L): 0.23
44	*Klebsiella*<br>*EBV*	Vancomycin<br>Fluconazole<br>Imipenem	*Neisseria* (throat swab)	Sepsis<br>Hodgkin lymphoma<br>Stomatitis	WBC (10^9/L):0.3<br>CRP (mg/L):90<br>PCT (μg/L): <0.02
47	*Klebsiella*	Sulperazon<br>Fluconazole<br>Ornidazole	*Neisseria* (throat swab)<br>*S. viridans* (throat swab)	Pneumonia<br>Stomatitis<br>ALL	WBC (10^9/L):2.13<br>CRP (mg/L): <5<br>PCT (μg/L): 0.1
48	*Streptococcus*	Sulperazon	*Enterobacteriaceae* (faeces)	Sepsis<br>Intestinal infection<br>ALL	WBC (10^9/L): -<br>CRP (mg/L): -<br>PCT (μg/L): -
50	*A. Baumanii*<br>*Streptococcus*	Meropenem<br>Vancomycin<br>Fluconazole<br>Azithromycin	*Serratia marcescens* (gastric juice)<br>*Enterobacteriaceae* (faeces)	Sepsis<br>Septic shock<br>Severe pneumonia<br>B-cell lymphoma	WBC (10^9/L):5.97<br>CRP (mg/L):165<br>PCT (μg/L): 0.48
52	*S. aureus*	Meropenem<br>Vancomycin	-	Sepsis<br>Intestinal infection<br>Neuroblastoma	WBC (10^9/L):0.14<br>CRP (mg/L): >160<br>PCT (μg/L): 11.71
56	*Klebsiella*	Imipenem and Cilastatin Sodium	-	Sepsis<br>Pneumonia	WBC (10^9/L):14.57<br>CRP (mg/L): 23

		Isopamicin<br>Meropenem<br>Vancomycin<br>Azithromycin<br>Linezolid<br>Cefatriaxone<br>Fluconazole		B-cell lymphoma	PCT (μg/L): <0.05
58	*Klebsiella*<br>*EBV*<br>*CMV*	Sulperazon<br>Fluconazole<br>SMZ<br>Voriconazole<br>Ornidazole<br>Imipenem and Cilastatin Sodium<br>Isopamicin<br>Tigecycline	-	Pneumonia<br>Acute cholecystitis<br>ALL	WBC (10^9/L):3.68<br>CRP (mg/L): 15<br>PCT (μg/L): 0.1
60	*Klebsiella*	Sulperazon<br>Vancomycin	*Enterococcus* (faeces)	Sepsis<br>RTI<br>Embryonal rhabdomyosarcoma	WBC (10^9/L):1.38<br>CRP (mg/L): 102<br>PCT (μg/L): 1.63
64	*E. Coli*	Meropenem<br>Vancomycin	*S. viridans* (sputum)	Sepsis<br>Pneumonia<br>Rhabdomyoid tumor	WBC (10^9/L):27.71<br>CRP (mg/L):87<br>PCT (μg/L): 3.61
73	*Enterococcus*<br>*EBV*	Sulperazon<br>Meropenem<br>Voriconazole	*S. viridans* (throat swab)<br>*Enterococcus* (faeces)	Pneumonia<br>Intestinal infection<br>ALL	WBC (10^9/L):2.2<br>CRP (mg/L): <5<br>PCT (μg/L): 0.14

75	*Enterococcus*	SMZ<br>Sulperazon<br>Meropenem	*Enterobacteriaceae* (faeces)	Chronic superficial gastritis (CSG)<br>ALL<br>RTI	WBC (10^9/L):2.15<br>CRP (mg/L): 133<br>PCT (μg/L): 0.54
**Presumptive false-positive**					
25	*P. Aeruginosa*<br>*E. Coli*	Cefuroxim	*E. faecalis* (urine)<br>*E. coli* (urine)<br>*Enterobacteriaceae* (faeces)<br>*Enterococcus* (faeces)	Systemic lupus erythematosus (SLE)<br>Lupus nephritis	WBC (10^9/L):6.06<br>CRP (mg/L): <5<br>PCT (μg/L): -

Interference Test

Validation Protocol

(1) The substances and concentrations of interferences.

Interfering substance	Final concentration of sample (mg/ml)
Human hemoglobin	20
Human ferroheme	0.2

Positive templates of the pathogen DNA mix according to P1, P2, P3, P4 and P5 were diluted to 50 copies/μl using distilled water, respectively.

(2) Verification method:

a). Verification of human hemoglobin: 2 ml negative plasma was taken, and added into 3 μl (150 copies) positive template P1 or P2, P3, P4, P5 and 20 mg/ml human hemoglobin, respectively. The detection was carried out for 5 times following the manufacturer's instruction.

b). Verification of human ferroheme: 2 ml negative plasma was obtained and added to 150 copies (3 μl) positive template P1 or P2, P3, P4, P5, and 0.2 mg/ml of human ferroheme. The detection was performed for 5 times following the manufacturer's instruction.

Diagnostic Criteria

Repeated detection for five times, and the positive rate of each target is 100%.

Results

The positive rate of the target was 100% for each ddPCR panel (**Figure S1**).

Figure S1. The results of interference test using human hemoglobin and human ferroheme. a). The verification test of interference factors on Panel 1; b). The verification test of interference factors on Panel 2; c). The verification test of interference factors on Panel 3; d). The verification test of interference factors on Panel 4; e). The verification test of interference factors on Panel 5. ROX, ROX channel; VIC, VIC channel; FAM, FAM channel; CY5, CY5 channel; A425, A425 channel.

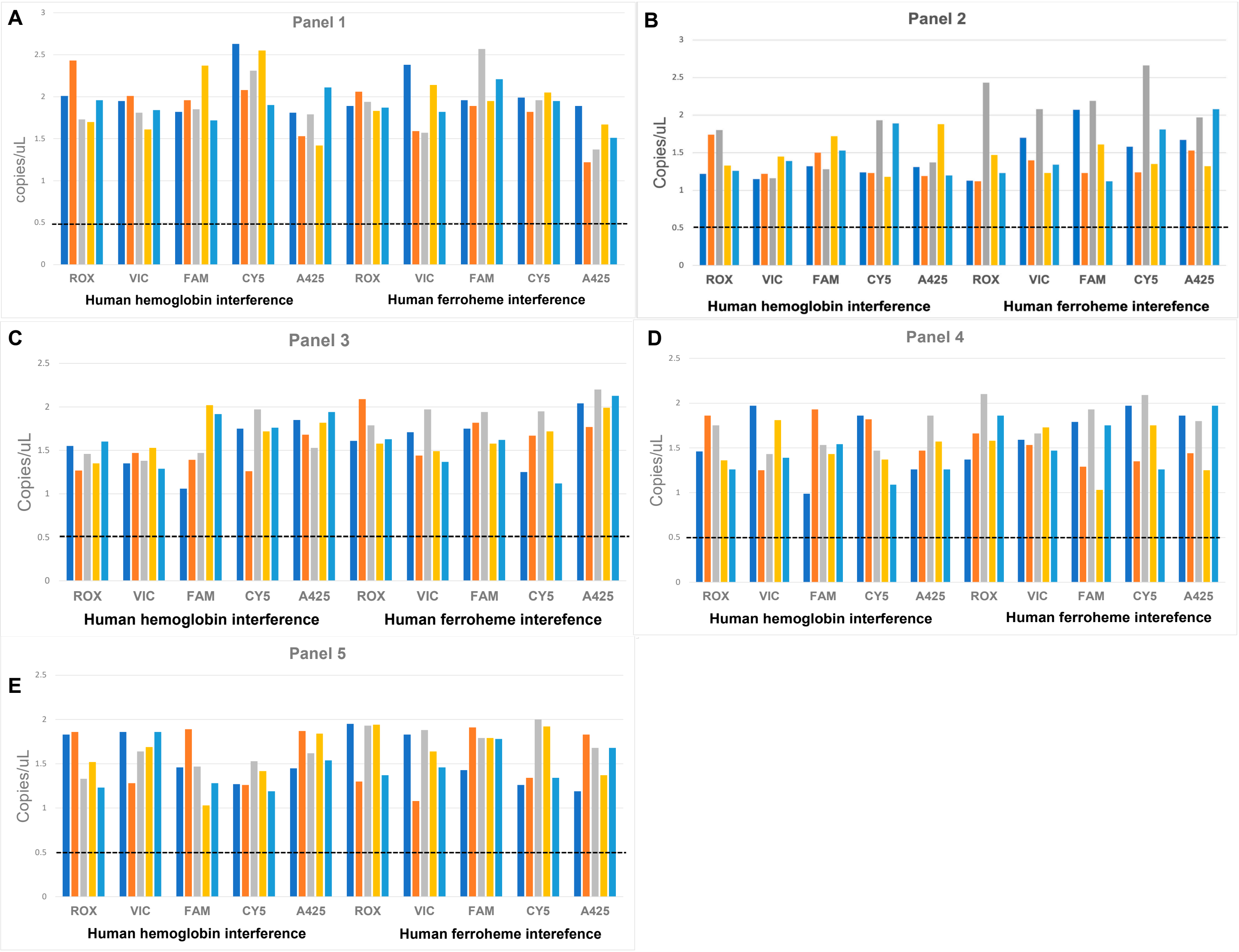
A
Panel 1
copies/uL
ROX
VIC
FAM
CY5
A425
Human hemoglobin interference
Human ferroheme interference
B
Panel 2
Copies/uL
Human hemoglobin interference
Human ferroheme interefence
C
Panel 3
Copies/uL
Human hemoglobin interference
Human ferroheme interefence
D
Panel 4
Copies/uL
Human hemoglobin interference
Human ferroheme interefence
E
Panel 5
Copies/uL
Human hemoglobin interference
Human ferroheme interefence

Conclusion

The detection performance of ddPCR panels were not interfered by with the mixing of human hemoglobin and ferroheme.

Cross-validation Test

Validation Protocol

(1) sample:

a). Positive template P1: DNAs from the standard strains of *Pseudomonas aeruginosa*, *Escherichia coli*, *Klebsiella pneumoniae* and *Acinetobacter baumannii* were extracted and diluted, respectively. Then, the DNAs were mixed and labeled as P1.

b). Positive template P2: DNAs from the standard strains of *Staphylococcus aureus, Candida albicans, Enterococcus faecium, Streptococcus pneumoniae and Staphylococcus epidermidis* were extracted and diluted, respectively. Then, the DNAs were mixed and labeled as P2.

c). Positive template P3: The synthetic positive plasmids containing *KPC*, *mecA*, *OXA-48*, *NDM*, *IMP*, *vanA*, *vanM* genes were diluted, mixed together, and labeled as P3.

d). Positive template P4: DNAs from the standard strains of *Salmonella, Bacteroides fancilis, Listeria monocytogenes, Haemophilus influenzae and*

Morganella mosani were extracted and diluted, respectively. Then, the DNAs were mixed and labeled as P4.

e). Positive template P5: DNAs from the standard products of HSV-1, VZV, EBV, CMV, HSV-2 were extracted and diluted. All nucleic acids were mixed together and labeled as P5.

(2) Verification method:

Panel 1-5 was used to detect all five positive templates P1, P2, P3, P4 and P5, and the no template control (NTC), respectively.

Diagnostic Criteria

P1/P2/P3/P4/P5：FAM channel/VIC channel/ROX channel/CY5 channel/A425 channel＜0.5 copies/μl，CY5.5 channel≥50 copies/μl;

Negative quality control products：FAM channel/VIC channel/ROX channel/CY5 channel/A425 channel＜0.5 copies/μl，CY5.5 channel≥50 copies/μl;

Positive quality control products：FAM channel/VIC channel/ROX channel/CY5 channel/A425 channel≥0.5 copies/μl，CY5.5 channel≥50 copies/μl;

Results

The ddPCR panels detected their corresponding target template, while no signal was observed from the other template DNAs (**Figure S2**).

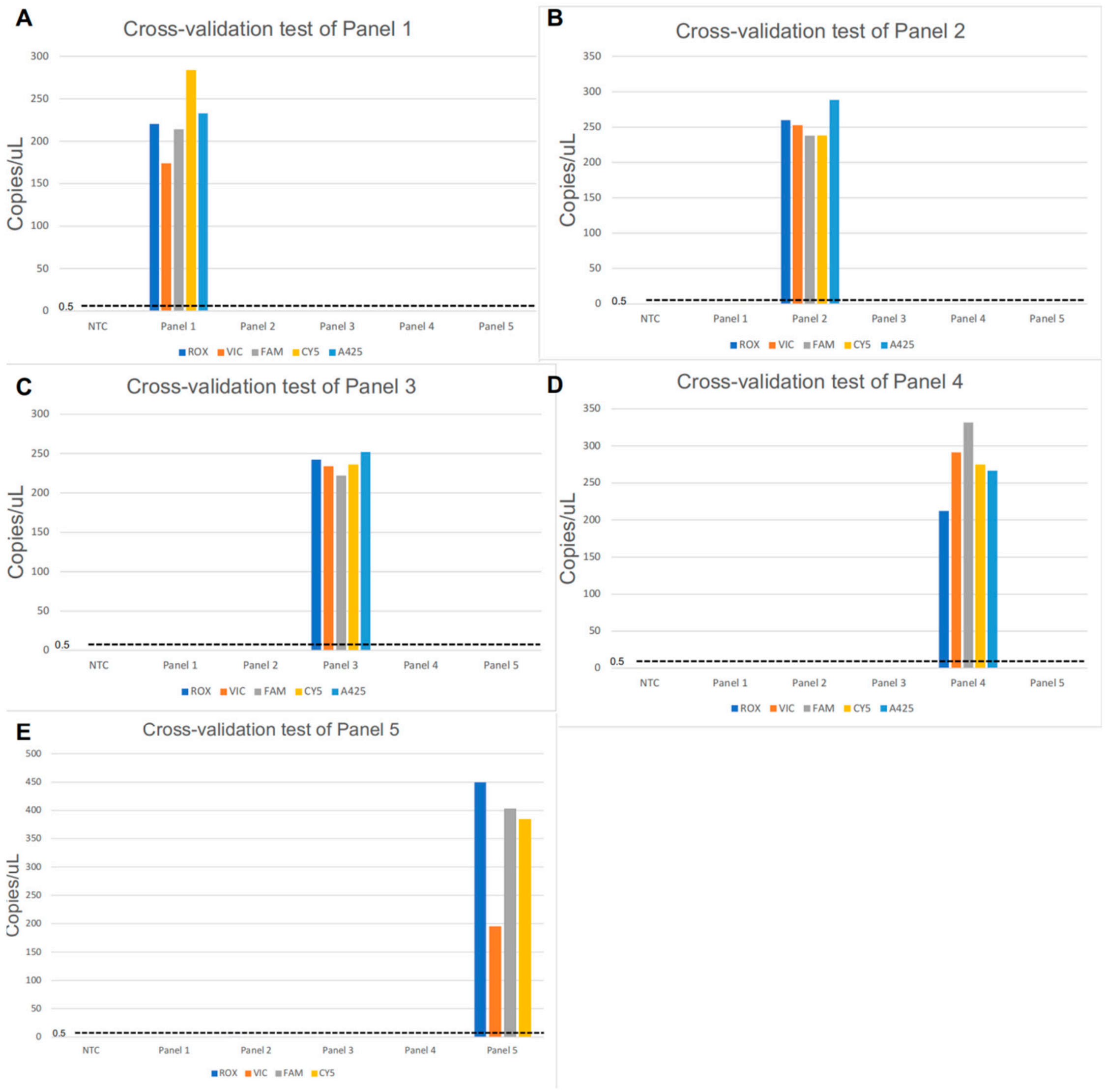
A
Cross-validation test of Panel 1
Copies/uL
B
Cross-validation test of Panel 2
Copies/uL
C
Cross-validation test of Panel 3
Copies/uL
D
Cross-validation test of Panel 4
Copies/uL
E
Cross-validation test of Panel 5
Copies/uL
0.5
NTC
Panel 1
Panel 2
Panel 3
Panel 4
Panel 5
ROX
VIC
FAM
CY5
A425

Figure S2. The results of cross-validation test. a). Positive template P1 verified other four panels; b). Positive template P2 verified other four panels; c). Positive template P3 verified other four panels; d). Positive template P4 verified other four panels; e). Positive template P5 verified other four panels. ROX, ROX channel; VIC, VIC channel; FAM, FAM channel; CY5, CY5 channel; A425, A425 channel.

Conclusion

The ddPCR panels can specifically detect their target without cross-reaction.
